# [^18^F]Fluoride PET provides distinct information on disease activity in ankylosing spondylitis as compared to MRI and conventional radiography

**DOI:** 10.1007/s00259-022-06080-5

**Published:** 2022-12-12

**Authors:** Jerney de Jongh, Nicki J. F. Verweij, Maqsood Yaqub, Christiaan J. van Denderen, Irene E. van der Horst-Bruinsma, Joost C. J. Bot, Bouke J. H. Boden, Robert Hemke, Frank F. Smithuis, Willem F. Lems, Adriaan A. Lammertsma, Alexandre E. Voskuyl, Maarten Boers, Gerben J. C. Zwezerijnen, Conny J. van der Laken

**Affiliations:** 1 Amsterdam Rheumatology and Immunology Center (ARC), Amsterdam UMC, Amsterdam, the Netherlands; 2 Department of Radiology & Nuclear Medicine, Amsterdam UMC, location VUmc, Amsterdam, the Netherlands; 3 Amsterdam Rheumatology and Immunology Center (ARC), location Reade, Amsterdam, the Netherlands; 4 Radboud University Medical Center, Rheumatology, Nijmegen, the Netherlands; 5 OLVG, Radiology & Nuclear Medicine, Amsterdam, the Netherlands; 6 Radiology & Nuclear Medicine, Amsterdam UMC, location AMC, Amsterdam, the Netherlands; 7 Epidemiology & Data Science, Amsterdam UMC, Vrije Universiteit, Amsterdam, the Netherlands

**Keywords:** Ankylosing spondylitis, Imaging, PET, MRI, [^18^F]Fluoride

## Abstract

**Purpose:**

To relate [^18^F]fluoride uptake on PET with abnormalities on magnetic resonance imaging (MRI) and conventional radiography (CR) in ankylosing spondylitis (AS) patients.

**Methods:**

Ten clinically active AS patients (female 6/10, age 38 ± 11 years) were included, and both spine and SI-joints were examined. PET scans were dichotomously scored for enhanced [^18^F]fluoride uptake, MRI scans were scored for fatty lesions, erosions, ankylosis, and bone marrow edema (BME), and CR was scored for erosions, syndesmophytes, and ankylosis. The overlap of lesions across all modalities was evaluated through univariate and multivariate analyses using a generalized mixed model.

**Results:**

In the spine, 69 lesions with enhanced [^18^F]fluoride uptake, 257 MRI lesions, and 88 CR lesions were observed. PET lesions were mostly located in costovertebral and facet joints, outside the field of view (FOV) of the MRI and CR. However, PET lesions inside the FOV of MRI and CR partially showed no abnormality on MRI and CR. In lesions with abnormalities on multiple modalities, both univariate and multivariate analysis showed that PET activity had the strongest association with BME on MRI and ankylosis on CR. In the SI joints, 15 lesions (75%) with PET uptake were found, with 87% showing abnormalities on MRI and CR.

**Conclusion:**

[^18^F]fluoride PET lesions are often found outside the scope of MRI and CR, and even in the same location show only partial overlap with abnormalities on MRI (especially BME) and CR (especially ankylosis). This suggests that [^18^F]fluoride PET partially visualizes aspects of AS separate from MRI and CR, providing novel information.

**Clinical trial registration:**

NL43223.029.13 registered at 02-05-2013. https://www.toetsingonline.nl/to/ccmo_search.nsf/fABRpop?readform&unids=C1257BA2002CC066C1257B4E0049A65A

**Supplementary Information:**

The online version contains supplementary material available at 10.1007/s00259-022-06080-5.

## Introduction

Bone formation is a hallmark of ankylosing spondylitis (AS), as it causes structural damage through the formation of syndesmophytes and spinal ankylosis [1]. Although historically, bone formation was thought to be a mere response to inflammation, research over the last two decades has demonstrated that new bone formation also occurs in the absence of inflammation [2, 3]. It still needs to be established whether the bone formation is related to some sort of repair mechanism following inflammation or whether it actually represents a completely separate pathway independent of inflammation.

In clinical practice, conventional radiography (CR) is used to detect bone formation in the axial skeleton, but it is only able to visualize pathology after several years [4, 5]. In contrast, a recent report suggests that [^18^F]fluoride positron emission tomography (PET) can show new bone formation in AS, up to 2 years before CR [6]. Previous data suggest that AS activity on PET is reflected by bone formation rather than inflammation, making [^18^F]fluoride more suitable than fluorodeoxyglucose (FDG) or macrophage tracers, and is reversible under effective therapy [7]. Decrease in molecular bone formation can be detected as early as 12 weeks after the start of anti-TNF treatment, which offers opportunities for early evaluation on treatment response.

Magnetic resonance imaging (MRI) is a highly sensitive technique for imaging inflammatory activity in AS by showing bone marrow edema (BME) and/or enhanced gadolinium uptake [8, 9]. In addition to imaging inflammation, MRI can also visualize both fatty changes and structural damage in the axial skeleton [10]. Although the relationship between bone formation and inflammation has previously been assessed, its exact nature remains unclear [3]. In addition, it remains to be determined how [^18^F]fluoride PET assessment of bone formation relates to inflammatory and structural changes on MRI and CR, and whether it has additional value to the assessment of AS compared to these clinically used imaging modalities.

The purpose of the present study was to perform a direct comparison of [^18^F]fluoride uptake in the axial column and SI-joints of AS patients and corresponding MRI and CR findings.

## Material and methods

### Patients and clinical assessment

Ten patients with clinically active AS were included. Patients (> 18 years) were included if they fulfilled the modified New York (mNY) criteria [11], had a Bath Ankylosing Spondylitis Disease Activity Index (BASDAI) of at least 4 [12], and had not previously been treated with biologicals. Patients were excluded if they had taken experimental drugs in the previous 3 months, or if they were either pregnant or breastfeeding. Clinical activity was assessed with inflammation markers such as the erythrocyte sedimentation rate (ESR) and C-reactive protein (CRP), and patient-reported outcome measures such as BASDAI [12], Bath Ankylosing Spondylitis Functional Index (BASFI) [13], Bath Ankylosing Spondylitis Metrology Index (BASMI) [14], and Ankylosing Spondylitis Disease Activity Score (ASDAS) [15]. Patients were allowed to continue the use of NSAIDS, provided they were on a stable dose.

The Medical Ethics Review Committee of the VU University Medical Center approved the study protocol. All patients gave informed consent prior to participation in the study.

### [^18^F]Fluoride PET scanning

[^18^F]Fluoride PET/CT scans were performed using either a Gemini TF-64 or an Ingenuity TF-128 PET/CT scanner (Philips Healthcare, Best, the Netherlands). Patients were injected with 100 ± 4 MBq [^18^F]fluoride through a venous cannula in the elbow, after which the intravenous catheter was flushed with 20 mL NaCl 0.9%. Residual activity was measured to accurately determine the amount of radioactivity injected. Forty-five minutes after injection, a low dose 30 mAs CT scan was performed, followed by a whole body PET scan from the base of the skull to the pelvis (including spine and SI-joints) using 5 min per bed position. Patients were scanned in supine position, with their hands placed on their laps.

Scan data were corrected for decay, scatter, randoms, and photon attenuation using standard procedures [16]. Following reconstruction, images were transferred to offline workstations for visual analysis. Static images of both spine and SI-joints were assessed visually by two experienced readers (OS and PR), blinded to other imaging and clinical data. Images were dichotomously scored as elevated or no elevated uptake, using background uptake in the vertebrae as reference. In case of disagreement, re-assessment by both readers was performed in order to reach consensus. Visual assessment was performed with standard 3D image viewing software, with the low dose CT as anatomical reference.

### MRI scanning

Gadolinium-enhanced MRI scans were performed using a Siemens Magnetom Sonata 1.5 Tesla scanner (Siemens Medical Solutions, Erlangen, Germany) within 1 week before or after the [^18^F]fluoride PET/CT scans. T1-weighted images, together with a short τ inversion recovery (STIR) sequence of the vertebral column and SI joints were acquired using head/neck and spine array, respectively. The technical details of the protocol conformed to the recommendations of the European Skeletal Society of Radiology (ESSR), as previously described [17].

Both STIR and T1-weighted images without gadolinium were assessed at the same time, by two experienced radiologists (RH and FS), who were blinded to clinical and other imaging data. All images of the spine were assessed for the presence of active inflammatory lesions in terms of subchondral BME, subchondral fatty marrow infiltration, ankylosis, syndesmophytes, and peri-articular erosions. Ankylosis or syndesmophyte formation, either caudal or cranial oriented, was only scored if present. In case of disagreement, re-assessment was performed in order to reach consensus. STIR and T1-weighted images of the SI-joints were assessed for the presence of erosions, ankylosis, and BME. Subchondral fatty marrow infiltration was not scored for SI-joints, as it was not possible to discriminate between sclerosis and fatty deposits due to the limited MRI settings used in the present study protocol.

### Conventional radiographs

Conventional radiographs of the complete (i.e., cervical, lumbar, and thoracic) spine and SI joints were performed, both in coronal and sagittal directions, in a window of 3 months before to 1 week after the PET/CT scan. Images were assessed visually and scored by two independent and experienced readers (IvdHB and CvD), who were blinded to clinical and other imaging data. In case of disagreement, radiographs were re-assessed in order to reach consensus. To assess the spine, the modified Stoke Ankylosing Spondylitis Spinal Score (mSASSS) was used. Lower and upper edges of each vertebrae were classified as normal, erosions/sclerosis/squaring, non-bridging syndesmophyte, or ankylosis [18]. Although this system has not been validated in the thoracic spine, it was used in the absence of an alternative. SI-joints were assessed according to the mNY criteria, grading 0–4 for each SI-joint [11].

### Assessment of lesions among imaging modalities

PET scans of the spine were assessed for activity at the following locations: processus spinosus, costovertebral joints, facet joints, and both anterior and posterior sites of the vertebrae. When comparing PET lesions with MRI lesions in the spine, only PET lesions located within the MRI field of view (FOV), at anterior, and posterior sites of the vertebrae, were included. PET lesions outside the MRI FOV were, for example, located at the processus spinosus, costovertebral, or facet joints. For the comparison with CR lesions, only lesions within the CR FOV, located at the anterior site of the vertebrae, were included. PET lesions outside the CR FOV were those located at the posterior site of a vertebra, the processus spinosus, costovertebral, and facet joints. PET scans of the SI-joints were assessed for activity in both joints separately, as were comparisons with MRI and CR.

### Statistical analysis

Clinical data and visual interpretation of PET, MRI, and CR data were evaluated using descriptive statistics. Cohen’s kappa scores were calculated over initial scores prior to consensus in order to derive inter-observer variability. In addition, the agreement between visual PET scores and various MRI and CR measures was determined using Cohen’s kappa. To assess the possible association between PET activity and different types of lesions on MRI and CR, and to take the multilevel structure of the data into account, a generalized linear mixed model with binominal distribution and logit link was used. The dichotomous PET outcome was included as the target and the patient was included as random effect. A nominal 5% level of significance was used, and only active PET lesions located within the FOV of the other modalities were included. First, univariate analysis was performed for all MRI and CR variables. Next, a multilevel analysis was performed including all MRI and CR variables as separate fixed effects. The model was reduced by manual steps, removing the least-significant interaction at each step through backward selection. Finally, a multilevel analysis was performed including MRI fat and MRI BME measures as separate fixed effects. SPSS version 28.0 software (SPSS, Chicago, IL, USA) was used to assess the distribution of both clinical and imaging data. A *P*-value smaller than 0.05 was considered to be significant.

## Results

### Clinical data

Patients had established disease, with an average duration of 7.0 ± 10.2 years since the initial diagnosis. Disease activity was high as assessed by BASDAI, ASDAS, BASMI, and BASFI (Table 1). PET/CT scans, MRI scans, and CR were obtained for all 10 patients. Due to technical issues, one thoracic CR could not be assessed for the presence of lesions.

**Table 1:** Patient characteristics. Numbers are count (%) or mean ± S.D. unless otherwise indicated

Females, count (%)	6 (60%)
HLA-B27 positive, number (%)	9 (90%)
Age, years (mean ± SD)	38 ± 10.9
Disease duration since diagnosis, years (mean ± SD)	7.0 ± 10.2
Duration of symptoms, years (mean ± SD)	10.3 ± 10.4
BASDAI (0–10), mean (S.D.)	5.2 (0.9)
ASDAS (0–10), mean (S.D.)	3.3 (0.5)
BASMI (0–10), mean (S.D.)	2.7 (1.8)
BASFI (0–10), mean (S.D.)	3.4 (1.9)
CRP, median (IQR), mg/mL	11.4 (11.0)
Patient global disease activity (0–10), mean (S.D.)	6.3 (1.3)
Treatment type	
NSAIDs, number (%)	6 (60)
Coxibs, number (%)	4 (40)

### Visual findings on all imaging modalities

#### PET/CT

**Fig. 1:**
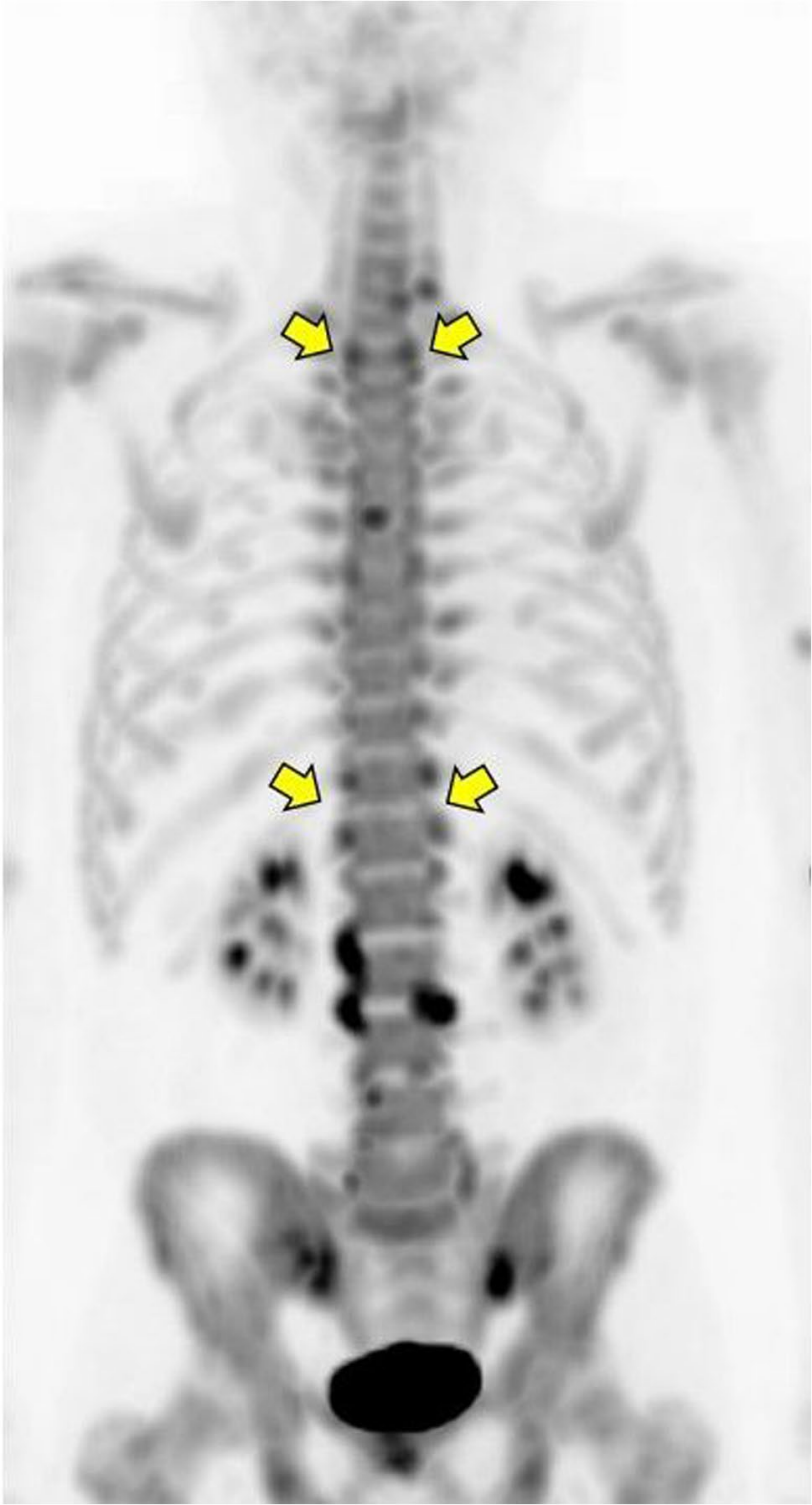
Elevated [^18^F]fluoride uptake in the costovertebral joints

In the spine, 6 out of the 10 patients showed PET activity at one or more anatomical locations. The number of spinal PET lesions per patient ranged from 2 to 19, with a total of 69 spinal lesions in all 10 patients. PET lesions were frequently located in the costovertebral or facet joints (example in Fig. 1). In the SI-joints, 9 patients showed PET enhancement in one or both joints, with a total of 15 SI-joints across patients.

#### MRI

MRI abnormalities (*n* = 257) were found in all patients. On MRI, a total of 64 lesions in seven patients, showed a combination of two or three abnormalities at the same spinal location. The following combinations were observed; fatty and ankylosis lesions (55%), BME en ankylosis lesions (39%), fatty and BME lesions (2%), and fatty, ankylosis and BME lesions (5%). All patients showed abnormalities in one or both SI-joints. On MRI, a total of 35 different MRI lesions (including combinations of lesion types) were found, which consisted of erosive lesions (43%), BME lesions (40%), and ankylosis lesions (17%). Seven patients showed a combination of two or three lesion types in one or both SI-joints, i.e., erosive and BME lesions (67%) and erosive, ankylosis, and BME lesions (33%).

#### CR

Spinal CR abnormalities (*n* = 83) were found in 8 patients, whereas all patients showed abnormalities in one or both SI-joints (Table 3). The frequency at the lesion level for all 3 modalities is outlined in Tables 2 and 3. All patients showed abnormalities in one or both SI-joints, with distribution at the lesion level outlined in Table 3.

**Table 2:** Overview of different types of lesions per spine segment for each modality. All lesions are included, regardless of their position within or outside the FOV of other modalities

Joint level	Cervical spine	Thoracic spine	Lumbar spine	Total
PET total, *n* (%)	4 (6)	42 (61)	23 (33)	69 (100)
MRI–fatty lesions, *n* (%)	15 (25)	30 (50)	15 (25)	60 (100)
MRI–erosions, *n* (%)	0 (0)	0 (0)	1 (100)	1 (100)
MRI–ankylosis, *n* (%)	2 (1)	121 (81)	27 (18)	150 (100)
MRI–BME, *n* (%)	1 (2)	35 (76)	10 (22)	46 (100)
CR erosions/sclerosis/squaring, *n* (%)	14 (36)	25 (64)	0 (0)	39 (100)
CR non-bridging syndesmophytes, *n* (%)	1 (50)	0 (0)	1 (50)	2 (100)
CR ankylosis, *n* (%)	4 (10)	31 (74)	7 (17)	42 (100)

**Table 3:** Overview of different types of lesions per SI-joint on all modalities

Joint level	SI-joint left	SI-joint right	Total
PET total, *n* (%)	7 (47)	8 (53)	15 (100)
MRI–erosions, *n* (%)	8 (53)	7 (47)	15 (100)
MRI–ankylosis, *n* (%)	3 (50)	3 (50)	6 (100)
MRI–BME, *n* (%)	5 (36)	9 (64)	14 (100)
CR mNY grade 0, *n* (%)	1 (100)	0 (0)	1 (100)
CR mNY grade 1, *n* (%)	1 (100)	0 (0)	1 (100)
CR mNY grade 2, *n* (%)	1 (14)	6 (86)	7 (100)
CR mNY grade 3, *n* (%)	6 (67)	3 (33)	9 (100)
CR mNY grade 4, *n* (%)	1 (50)	1 (50)	2 (100)

The inter-observer variability before consensus for all imaging modalities is summarized in Supplementary Table 1. Associations between consensus scores of the various imaging modalities were low, as illustrated by the Cohen’s kappa scores (Supplementary Table 2).

### Comparison between imaging modalities

In total, 45 and 46 out of the 69 lesions PET lesions were located outside the FOV of MRI and CR, respectively. PET lesions were frequently located in the costovertebral (49% and 48% for MRI and CR, respectively) or facet (20% for both MRI and CR) joints. A lack of any type of abnormalities on both MRI and CR was found in 5 PET lesions.

### Association between [^18^F]fluoride PET and MRI

**Fig. 2:**
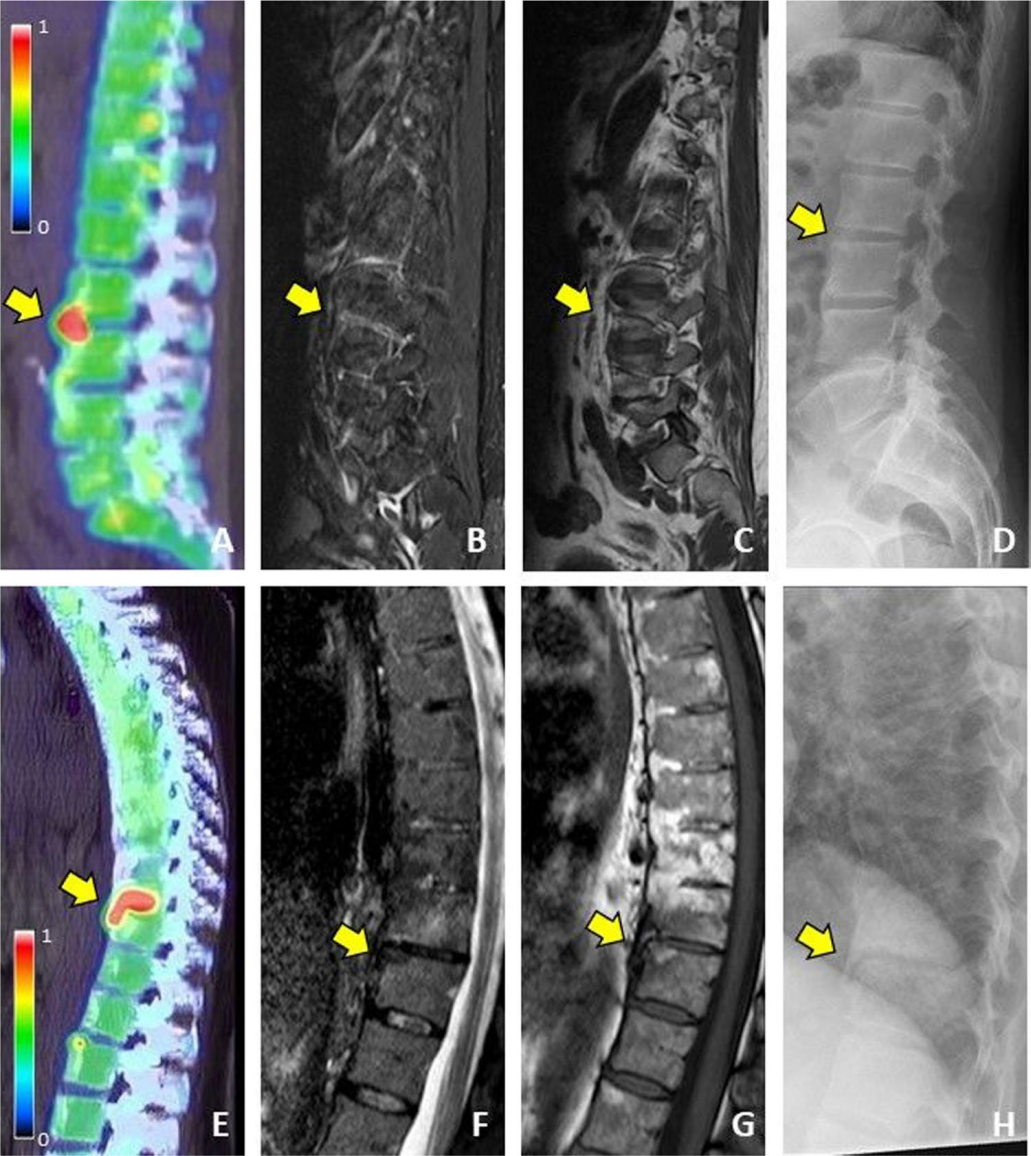
Lumbar spine of a female AS patient showing [^18^F]fluoride uptake on PET (**A**), no BME on MRI STIR (B^*^), no fat infiltration on MRI T1 (C^*^), and no abnormalities on CR (**D**), and thoracic spine of another female AS patient showing [^18^F]fluoride uptake on PET (**E**), minimal BME on MRI STIR (**F**), minimal fat infiltration on MRI T1 (**G**), and non-bridging syndesmophyte formation on CR (**H**).^*^The position of the image is caused by the lateral location of the PET lesion

**Fig. 3:**
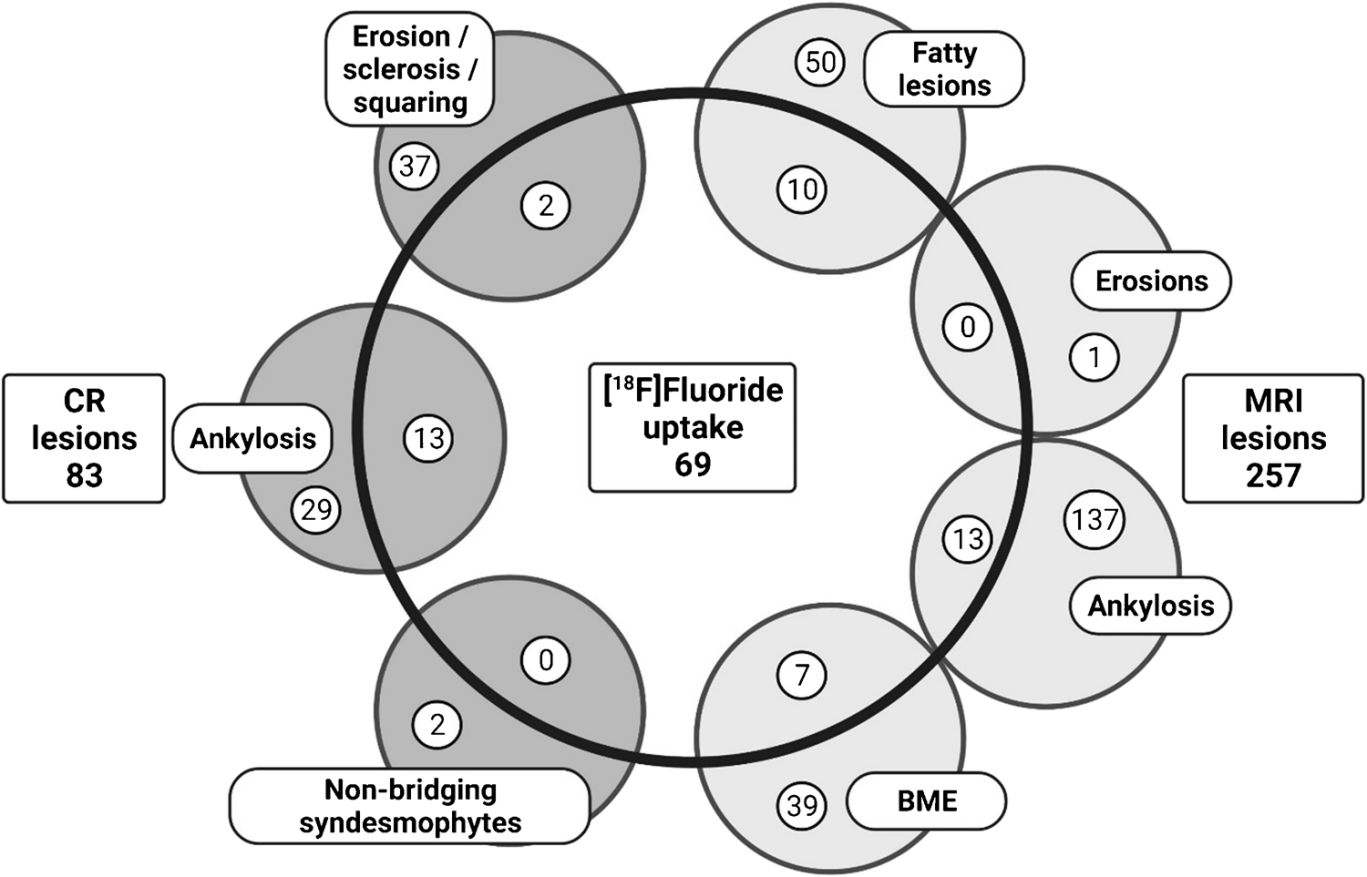
Overlap between lesions on different modalities in the vertebral spine

In the spine, twenty-four (out of 69, 35%) PET active sites were located within the FOV of MRI (example shown in Fig. 2). Half of these lesions were located in the lumbar spine and, in general, they were positioned at the anterior part of the vertebral units (96%). Seventeen out of these 24 lesions also showed abnormalities on MRI, with 10 (43%), 13 (54%), and 7 (29%) showing fatty, ankylosis, and/or BME lesions, respectively. These findings are summarized in Fig. 3.

In the SI-joints, 13 out of 15 PET lesions (87%) also showed abnormalities on MRI. Erosions, ankylosis, and BME lesions were observed in 13 (87%), 4 (27%), and 13 (87%) of these lesions, respectively.

### Association between [^18^F]fluoride PET and CR

Twenty-three (out of 69, 33%) PET active sites were located within the FOV of CR. Most lesions (52%) were located in the lumbar spine, and 15 also showed abnormalities on CR, i.e., 2 (9%) and 13 (57%) with erosive and non-bridging syndesmophyte lesions, respectively. Increased PET uptake was seen in SI-joints that were scored as grade 1, grade 2, and grade 3 (7%, 33%, and 53%, respectively). One PET active SI-joint showed no abnormalities on CR.

### Association between [^18^F]fluoride PET, MRI, and CR

Univariate analysis showed that the presence of ankylosis lesions on CR was associated most strongly with PET activity, (odds ratio (OR) 34, 95% confidence interval (CI) 6–183, *p* < 0.01), followed by the presence of BME lesions on MRI (OR 6.6, 95% CI 2–21, *p* < 0.01). No other significant associations with increased PET uptake were identified in the univariate analysis, although the presence of fatty lesions on MRI showed a trend towards an association (OR 2.9, 95% CI 1–9, *p* = 0.06). These results are summarized in Supplementary Table 3.

Multivariate analysis including all MRI and CR lesions, except MRI erosion, and CR non-bridging syndesmophyte lesions, as these lesions were almost absent in the dataset, indicated that the combination of both MRI, BME, and CR ankylosis lesions provided the best association with PET activity. Using backward selection, the least significant variable was excluded, until MRI BME (OR 6.4, 95% CI 2–24, *p* = 0.005) and CR ankylosis (OR 39, 95% CI 7–223, *p* < 0.001) remained, confirming the results of the univariate analysis. Multivariate analysis including MRI fat and MRI BME lesions showed that the combination of these two variables was significantly associated with PET activity (MRI fat; OR 3.3, 95% CI 1.05–10.6, *p* = 0.04 and MRI BME; OR 7, 95% CI 2.19–22.6, *p* < 0.01).

## Discussion

This is the first study in clinically active AS patients that combines the separate modalities of PET, MRI, and CR in the spine and SI-joints. The study demonstrates that molecular bone formation visualized on [^18^F]fluoride PET provides distinct information as compared with abnormalities shown on MRI and CR. The vast majority (roughly two out of three) of the PET lesions in this study were located outside the FOV of MRI and CR, in facet and costovertebral joints. More importantly, out of all spinal PET lesions that were located within the field of view of MRI and CR, 29 and 35% showed no correspondence with abnormalities on MRI and CR, respectively. For lesions in the spine that showed correspondence, [^18^F]fluoride uptake was most strongly associated with BME on MRI and ankylosis on CR. These findings suggest that molecular bone formation, as measured by [^18^F]fluoride PET, is associated with both inflammation and structural damage.

To date, the number of reports on head-to-head comparison of [^18^F]Fluoride PET and other imaging modalities is limited. Nevertheless, several studies have shown a trend toward an association between [^18^F]fluoride uptake and inflammatory changes, as depicted by BME, on MRI [19–22]. In addition, it has been suggested that fat infiltration on MRI is related with later development of focal bone formation [23]. Indeed, some studies only find an overlap between [^18^F]Fluoride uptake and fatty lesions on MRI, which was not present for BME [24]. Discrepant findings on the association of [^18^F]fluoride uptake and structural abnormalities on MRI or CT have been reported, with some studies describing an association with syndesmophyte formation [1, 6], whilst others failed to find a relationship [21, 22]. The findings of the present study are in concordance with most previous studies, suggesting a connection between bone formation as portrayed by [^18^F]fluoride uptake and both acute inflammation and structural changes, while also at least in part visualizing a separate process that remains outside the scope of MRI and CR. However, so far, cross-sectional observations have not clarified if, and, if so, how and when new bone formation on PET is linked to inflammation and structural damage [25]. This would require future longitudinal comparative studies between PET, MRI, and CR.

As mentioned above, most of the PET lesions in the present study were located outside the FOV of MRI and CR. The importance of imaging facet and costovertebral joints for the assessment of AS activity has been demonstrated previously [26]. Unfortunately, CR is unable to properly visualize these joints, as the vertebral spine cannot be imaged axially. In addition, although these joints can be included in the field of view of MRI, standard protocols focus on sagittal and transverse sequences and do not extend to costovertebral joints. It has been suggested that this approach could possibly miss 44% of inflamed vertebrae in the thoracic spine and 16% in the lumbar spine [27]. In previous comparative studies, [^18^F]fluoride PET depicted more lesions in active AS than MRI [17, 19]. This may, at least in part, be related to the whole-body imaging approach of PET as opposed to the limited field of view used in MRI studies, but it could also be related to the fact that both imaging modalities look at different aspects of the disease process. Nevertheless, the ability of [^18^F]fluoride PET to visualize lesions that are outside the FOV of routine MRI and CR studies, is a major advantage for assessing disease activity in AS.

A limitation of the present study is the relatively small sample size, which was due to the proof-of-concept character of the study. Despite the small number of lesions, however, both univariate and multivariate analyses provided statistically significant results. On the other hand, due to the limited numbers, univariate, and multivariate analyses were only possible on lesions in the vertebral column and not in the SI-joints. In addition, as almost no erosion or non-bridging syndesmophyte lesions were present on MRI and non-bridging lesions were nearly absent on CR, it remains unclear how these lesions would affect the relationship between [18F]fluoride PET and MRI or CR. Another limitation is the fact that at the time of inclusion it was not possible to assess AS activity on the low-dose CT, as it was not yet possible to perform the bone reconstruction. Although the low-dose CT images could potentially provide additional information to the dataset, they could unfortunately not be included in the current study.

## Conclusion

Although most PET lesions were found outside the FOV of MRI and CR, even PET lesions inside the FOV partially showed no abnormalities on MRI and CR. When comparing overlapping lesions, MRI, BME, or CR ankylosis abnormalities were most strongly associated with [^18^F]Fluoride uptake, indicating a relation between bone formation and inflammation on MRI as well as structural bone damage on CR. This confirms that [^18^F]fluoride PET provides distinct disease information. In addition, the larger FOV of whole-body PET is a major advantage over the more limited FOV of routine MRI and CR studies.

## Supplementary Information

Below is the link to the electronic supplementary material.

Supplementary file1 (DOCX 15 KB)

## Data Availability

The datasets generated and/or analyzed during the current study are available from the corresponding author on reasonable request.

## References

[1] Lee SG (2015). Assessment of bone synthetic activity in inflammatory lesions and syndesmophytes in patients with ankylosing spondylitis: the potential role of 18F-fluoride positron emission tomography-magnetic resonance imaging. Clin Exp Rheumatol.

[2] van der Heijde D (2012). MRI inflammation at the vertebral unit only marginally predicts new syndesmophyte formation: a multilevel analysis in patients with ankylosing spondylitis. Ann Rheum Dis.

[3] Baraliakos X (2008). The relationship between inflammation and new bone formation in patients with ankylosing spondylitis. Arthritis Res Ther.

[4] Forestier J, Deslous-Paoli P (1957). Radiological study of sacro-iliac joints in ankylosing spondylitis with reference to the evolution of the disease. Ann Rheum Dis.

[5] Rudwaleit M, Khan MA, Sieper J (2005). The challenge of diagnosis and classification in early ankylosing spondylitis: do we need new criteria?. Arthritis Rheum.

[6] Park EK (2017). Baseline increased 18F-fluoride uptake lesions at vertebral corners on positron emission tomography predict new syndesmophyte development in ankylosing spondylitis: a 2-year longitudinal study. Rheumatol Int.

[7] Bruijnen STG (2018). Bone formation in ankylosing spondylitis during anti-tumour necrosis factor therapy imaged by 18F-fluoride positron emission tomography. Rheumatology (Oxford).

[8] Maksymowych WP, Landewé R (2006). Imaging in ankylosing spondylitis. Best Pract Res Clin Rheumatol.

[9] Maksymowych WP, Lambert RG (2007). Magnetic resonance imaging for spondyloarthritis–avoiding the minefield. J Rheumatol.

[10] Hermann KG (2012). Descriptions of spinal MRI lesions and definition of a positive MRI of the spine in axial spondyloarthritis: a consensual approach by the ASAS/OMERACT MRI study group. Ann Rheum Dis.

[11] van der Linden S, Valkenburg HA, Cats A. Evaluation of diagnostic criteria for ankylosing spondylitis. A proposal for modification of the New York criteria*.* Arthritis Rheum. 1984; 27(4):361-8. 10.1002/art.1780270401. 10.1002/art.1780270401 6231933

[12] Garrett S (1994). A new approach to defining disease status in ankylosing spondylitis: the Bath Ankylosing Spondylitis Disease Activity Index. J Rheumatol.

[13] Calin A (1994). A new approach to defining functional ability in ankylosing spondylitis: the development of the Bath Ankylosing Spondylitis Functional Index. J Rheumatol.

[14] Jenkinson TR, et al. Defining spinal mobility in ankylosing spondylitis (AS). The Bath AS Metrology Index. J Rheumatol. 1994;21(9):1694-8. 7799351

[15] Lukas C (2009). Development of an ASAS-endorsed disease activity score (ASDAS) in patients with ankylosing spondylitis. Ann Rheum Dis.

[16] Boellaard R, Hoekstra OS, Lammertsma AA. Software tools for standardized analysis of FDG whole body studies in multi-center trials. J Nucl Med. 2008;49(no. supplement 1):159P.

[17] Bruijnen ST (2012). Bone formation rather than inflammation reflects ankylosing spondylitis activity on PET-CT: a pilot study. Arthritis Res Ther.

[18] Creemers MC (2005). Assessment of outcome in ankylosing spondylitis: an extended radiographic scoring system. Ann Rheum Dis.

[19] Buchbender C (2015). Hybrid 18F-labeled fluoride positron emission tomography/magnetic resonance (MR) imaging of the sacroiliac joints and the spine in patients with axial spondyloarthritis: a pilot study exploring the link of MR bone pathologies and increased osteoblastic activity. J Rheumatol.

[20] Sawicki LM (2018). Dual-phase hybrid (18) F-fluoride positron emission tomography/MRI in ankylosing spondylitis: investigating the link between MRI bone changes, regional hyperaemia and increased osteoblastic activity. J Med Imaging Radiat Oncol.

[21] Raynal M (2019). Performance of 18F-sodium fluoride positron emission tomography with computed tomography to assess inflammatory and structural sacroiliitis on magnetic resonance imaging and computed tomography, respectively, in axial spondyloarthritis. Arthritis Res Ther.

[22] Ouichka R (2019). Performance of 18F-sodium fluoride positron emission tomography with computed tomography to assess inflammatory and structural sacroiliitis on magnetic resonance imaging in axial spondyloarthritis. Clin Exp Rheumatol.

[23] Chiowchanwisawakit P (2011). Focal fat lesions at vertebral corners on magnetic resonance imaging predict the development of new syndesmophytes in ankylosing spondylitis. Arthritis Rheum.

[24] Fischer DR (2012). High bone turnover assessed by 18F-fluoride PET/CT in the spine and sacroiliac joints of patients with ankylosing spondylitis: comparison with inflammatory lesions detected by whole body MRI. EJNMMI Res.

[25] De Craemer ALZCP. Use of Imaging in Axial Spondyloarthritis for Diagnosis and Assessment of Disease Remission in the Year 2022*.* Curr Rheumatol Rep. 2022. 10.1007/s11926-022-01091-5. 10.1007/s11926-022-01091-5 36242738

[26] Chui ETF (2020). MRI inflammation of facet and costovertebral joints is associated with restricted spinal mobility and worsened functional status. Rheumatology (Oxford).

[27] Rennie WJ (2009). Magnetic resonance imaging assessment of spinal inflammation in ankylosing spondylitis: standard clinical protocols may omit inflammatory lesions in thoracic vertebrae. Arthritis Rheum.

