# Supplementary material for: [18F]Fluoride PET provides distinct information on disease activity in ankylosing spondylitis as compared to MRI and conventional radiography
Source: Eur J Nucl Med Mol Imaging. 2022 Dec 12;50(5):1351–9. doi: 10.1007/s00259-022-06080-5 (PMC10027810; doi:10.1007/s00259-022-06080-5)
Supplement: Supplementary file 1 — Supplementary file1 (DOCX 15 KB) [file 259_2022_6080_MOESM1_ESM.docx]

**Supplementary information**

**Article title:** [^18^F]Fluoride PET/CT adds novel information on disease activity in ankylosing spondylitis to MRI and conventional radiography

**Journal name:** European Journal of Nuclear Medicine and Molecular Imaging

**Author names:** J. de Jongh, N.J.F. Verweij, M. Yaqub, C.J. van Denderen, I.E. van der Horst-Bruinsma, J. Bot, B.J.H. Boden, R. Hemke, F.F. Smithuis, A.E. Voskuyl, M. Boers, G.J.C. Zwezerijnen, C.J. van der Laken

**Affiliation and e-mail address of the corresponding author:** Amsterdam UMC, Vrije Universiteit Amsterdam, Department of Rheumatology and Clinical Immunology, De Boelelaan 1117, Amsterdam, Netherlands, e-mail: [j.dejongh2@amsterdamumc.nl](mailto:j.dejongh2@amsterdamumc.nl)

|  | **Lesion type** | **κ** | ***P*** |
| --- | --- | --- | --- |
| Spine | PET | 0.45 | 0.00 |
|  | MRI - Fatty lesions | 0.84 | 0.00 |
|  | MRI - Ankylosis | 0.73 | 0.00 |
|  | MRI - BME | 0.72 | 0.00 |
|  | MRI - Erosions* | - | - |
|  | CR | 0.35 | 0.00 |
| SI-joints | PET | 0.77 | 0.00 |
|  | MRI - Ankylosis | 0.32 | 0.00 |
|  | MRI - BME | 0.50 | 0.00 |
|  | MRI - Erosions | 0.50 | 0.00 |
|  | CR | 0.53 | 0.00 |

Supplementary table 1: Inter-observer agreement on the PET, CR and initial MRI score (0-3). *Agreement for erosion lesions in the spine could not be calculated due to the limited amount of lesions

|  | **Lesion type** | **κ** | ***P*** |
| --- | --- | --- | --- |
| MRI score | Fatty lesions | 0.177 | <0.001 |
|  | Ankylosis | -0.004 | 0.814 |
|  | BME | 0.065 | 0.021 |
|  | Erosions | 0.141 | 0.001 |
| CR score | Erosions | 0.002 | 0.969 |
|  | Syndesmophytes | -0.008 | 0.745 |
|  | Ankylosis | 0.359 | <0.001 |

Supplementary table 2: Summary of all calculated kappa’s between PET score and MRI and CR score.

| **Variable** | **Coefficient** | **95% C.I. coefficient** | **Sig coefficient** | **Odds ratio** | **95% C.I. OR** |
| --- | --- | --- | --- | --- | --- |
| MRI ankylosis | 0.7 | -0.18 – 1.6 | 0.12 | 2.0 | 0.83 – 4.99 |
| **MRI BME** | **1.9** | **0.72 – 3.05** | **<0.01** | **6.6** | 2.05 – 21.1 |
| MRI erosions | -13.6 | -4729 – 4702 | 0.99 | 1.3 E-6 | 0.000 - ∞ |
| MRI fat | 1.1 | -0.03 – 2.16 | 0.06 | 2.9 | 0.97 – 8.69 |
| **CR ankylosis** | **3.5** | **1.84 – 5.21** | **<0.01** | **34** | 6.29 – 183 |
| CR erosions / sclerosis / squaring | 1.1 | -0.66 – 2.84 | 0.22 | 3.0 | 0.52 – 17.0 |
| CR non-bridging syndesmophytes | -14.6 | -5512 – 5482 | 0.99 | 4.6 E-7 | 0.000 - ∞ |

Supplementary table 3: Results of a univariate analysis for all independent MRI and CR variables with the PET variable as outcome.
